# Identification of the factors associated with outcomes in a Condition Management Programme

**DOI:** 10.1186/1471-2458-12-927

**Published:** 2012-10-30

**Authors:** Evangelia Demou, Iain Gibson, Ewan B Macdonald

**Affiliations:** 1 Institute of Health and Wellbeing, College of Medical, Veterinary and Life Sciences, University of Glasgow, 1 Lilybank Gardens, Glasgow, G12 8RZ, UK

**Keywords:** *Intervention*, V*ocational rehabilitation*, Incapacity benefit, Biopsychosocial model

## Abstract

**Background:**

A requirement of the Government’s *Pathways to Work* (PtW) agenda was to introduce a *Condition Management Programme* (CMP). The aim of the present study was to identify the differences between those who engaged and made progress in this telephone-based biopsychosocial intervention, in terms of their health, and those who did not and to determine the client and practitioner characteristics and programme elements associated with success in a programme aimed at improving health.

**Methods:**

Data were obtained from the CMP electronic spreadsheets and clients paper-based case records. CMP standard practice was that questionnaires were administered during the pre- and post-assessment phases over the telephone. Each client’s record contains their socio-demographic data, their primary health condition, as well as the pre- and post-intervention scores of the health assessment tool administered. Univariate and multivariate statistical analysis was used to investigate the relationships between the database variables. Clients were included in the study if their records were available for analysis from July 2006 to December 2007.

**Results:**

On average there were 112 referrals per month, totalling 2016 referrals during the evaluation period. The majority (62.8%) of clients had a mental-health condition. Successful completion of the programme was 28.5% (575 “completers”; 144 “discharges”). Several factors, such as age, health condition, mode of contact, and practitioner characteristics, were significant determinants of participation and completion of the programme. The results showed that completion of the CMP was associated with a better mental-health status, by reducing the number of clients that were either anxious, depressed or both, before undertaking the programme, from 74% to 32.5%.

**Conclusions:**

Our findings showed that an individual's characteristics are associated with success in the programme, defined as completing the intervention and demonstrating an improved health status. This study provides some evidence that the systematic evaluation of such programmes and interventions could identify ways in which they could be improved.

## Background

In response to the large increases in the incapacity benefit (IB) claimants which occurred in the ten years up to 1995, the Government introduced Pathways to Work (PtW) under the Welform Reform agenda to reduce the numbers receiving health-related benefits from 2.6 million in 2006/07 to 1.6 million or fewer by 2016/17 [1].

The lack of access to vocational rehabilitation and the absence of an integrated approach across health, benefit and employment agencies, could further support benefit-dependency [2]. The PtW scheme was intended to encourage employment among people claiming incapacity benefits [3]. A key aim of the initiative was to intervene early in an attempt to prevent welfare dependency, as one of the key predictors of remaining on benefit is time [3-5].

Most people who start claiming incapacity benefit do not report a severe health issue, and there are social and vocational obstacles which are associated with them staying on benefits [2,6]. Aside from the time spent on benefits, low confidence, beliefs that work will be harmful for their health, and financial hindrances when moving off benefits for low-paid employment are health and condition-independent factors that encourage remaining on benefits [2,6]. Multiple health problems, low income and unemployment are associated with reduced health-related quality of life in the working age population, whereas returning to a form of work can improve health and wellbeing [6-11].

A requirement of PtW was to introduce a *Condition Management Programme* (CMP) aimed at assisting claimants using a bio-psychosocial approach to better cope with their health condition, address the multiple factors influencing their wellbeing, and encourage positive thinking about health, lifestyle and work [12-14]. Participation in the programme was voluntary [12].

Many vocational rehabilitation programmes exist, but several review studies have found modest evidence that such programs actually work and support return-to-work [5,15-17]. Audhoe et al. (2010) demonstrated in a recent systematic review, that using group training techniques for workless individuals with mental health problems was not efficient in enhancing wellbeing [15]. The review of Riddell (2002) describes international vocational-rehabilitation programmes and their effects on return-to-work [5]. A six-country international study of workers, disabled by lower-back pain, using medical and non-medical interventions demonstrated no association between either intervention and return-to-work [5]. The authors even report a negative or neutral association between vocational rehabilitation efforts, in the form of training and education, with return-to-work within a one-year period [5]. Franche et al. (2004) reported that while return-to-work interventions were generally effective in reducing disability duration, wage replacement and health-care costs, the evidence was not clear that they were effective in enhancing quality of life [18]. Similarly, a Swedish study comparing chances of re-employment participating in rehabilitation programs versus no rehabilitation, found that many rehabilitation efforts have no positive effects on re-employment chances compared with no rehabilitation [19]. The review of Riddell (2002) also demonstrated that competitive work status at time of application and non-severe disability were good predictors of re-employment following participation in a rehabilitation programme, whilst receipt of benefits from government initiatives provided the lowest probabilities for competitive employment [5]. Additionally, it is recognised that the population of people claiming health related benefits reflects in part the state of the labour market, with some arguing that the number of benefit claimants is disguised unemployment [20,21]. Webster et al. (2010) suggest that the reduction in health related claimants is more closely related to the state of the labour market than any policies or initiatives for return-to-work [22].

Contrarily, some positive indication does exists [5,16]. The evaluation of a multi-disciplinary vocational-rehabilitation programme for unemployed people with back pain, who undertook a programme incorporating physical rehabilitation, psychological interventions and vocational advice and assistance, showed that for the two intervention groups that participated, six months after programme completion, 43% and 39% were employed, and a further 46% and 23% were participating in job training, education or voluntary work [5]. This study emphasized the need for suitable and multidisciplinary support across agencies to achieve successful outcomes [5]. The individual placement and support (IPS) approach has also been demonstrated to be effective in assisting clients with mental health problems in gaining or sustaining employment [23]. Rinaldi and Perkins (2007) have shown that the multidisciplinary approach of IPS, including its specialist employment expertise, successfully impacts upon the number of people gaining open employment and suggests that it could be as effective in sustaining employment for those at risk of unemployment due to their medical condition [23].

In Scotland there were around 300,000 IB clients in 2010 [24], with approximately 46,000 IB claimants in the region of Lanarkshire [24]. As part of PtW the CMP was implemented by NHS Lanarkshire, with the aim of utilising NHS services, and other services not routinely available via the NHS [12]. The programme was different from the traditional occupational health model, as its remit also included improving the client’s lifestyle and general attitude to life and work via a Cognitive Behavioural Therapy (CBT) approach [12]. The preferred CBT approach was the ‘5 areas approach’ pioneered by Williams (2001), which is known to be particularly successful in helping clients deal with assertiveness issues and anxiety/depression problems, and in helping clients regain ‘control’ of their lives [25]. The CBT components of the CMP were imbedded within a work-focussed programme delivered by experienced and trained practitioners over the telephone.

The aim of the present study was to identify the differences between those who engaged and made progress, in terms of their health, within the CMP and those who did not and to determine what were the client and practitioner characteristics and programme elements, which were associated with success in a programme aimed at improving health and potential for work.

## Methods

A client could be referred to the CMP if they met the standard criteria for PtW [6]. Condition Management was different from the traditional occupational health model, in that it included in its remit the possibility of changing and improving the client’s general approach to life and work. The core elements of the CMP programme are managing depression, anxiety, pain, fatigue, and addiction; promoting assertiveness; confidence building; coping strategies and lifestyle change [12].

The CMP administrator logged referrals that came from Jobcentre Plus and distributed cases to Condition Managers on a skills match basis. An aim of the CMP programme was that initial contact with the client occurs within five working days of referral, the initial assessment takes place no longer than five working days after this, and the client is started on an appropriate programme no longer than five working days after this. The process was administered on a one-to-one basis, on the telephone, over 4–14 weeks.

Following successful completion of the CMP, it is expected that the client (a) will be expected to understand more about their condition, will have developed strategies for dealing with their health condition and how to safely manage their condition in a workplace setting; (b) will have explored the health benefits of returning to work and will feel more confident about both returning to work and remaining in work when suitable employment has been found, and (c) will be in a better position to recognise the early signs of a relapse or worsening of their condition, and will be in a better position to take action to prevent this impacting on their employment.

Data were obtained from the CMP electronic spreadsheets and from clients paper-based case records. For this study, clients were divided into those who completed the programme-“completers”- and those who left the programme without completing for whatever reason-“discharges”. Clients were included in the study if their records were available for analysis from July 2006 to December 31^st^ 2007.

CMP standard practice was that questionnaires were administered during the pre- and post-assessment phases over the telephone. Each client’s record contains their socio-demographic data, their primary health condition for which they were receiving incapacity benefit, as well as the pre- and post-intervention scores of the assessment tools administered. The main assessment tool used was the Hospital Anxiety and Depression Scale (HADS), which detects the presence and severity of anxiety and depression and has previously been used in studies relating to health and unemployment [26]. The HADS questionnaire consists of 14 items and yields two measures, one for anxiety and one for depression. Scores on both scales can range between 0 and 21. A higher score indicates a more severe condition. A score of 7 or less is considered “normal”, a score between 8 and 10 “mild”, and a score above 11 indicates “caseness”, that is individuals would be considered anxious or depressed.

This study was an observational study using retrospective data and was a service evaluation. Clients were not recruited for this study and no data was collected outside of routine practice. All databases were anonymised. All clients referred into the CMP gave consent to their anonymised data being used for evaluation and auditing purposes. Consent was obtained at their first appointment.

### Statistical analysis

SPSS version 15 was used for the statistical analyses. Multivariate statistical analysis was used to investigate the relationships between the routinely collected CMP database variables and explore which predictor variables are associated with the outcome variable. The predictor variables were age, gender, deprivation, health condition, phone type, area, associated Jobcentre Plus, and Practitioner. For clients who completed the programme, sessions (i.e. # sessions attended), weeks (i.e. # weeks on programme), and elements (i.e. # modules undertaken) were also available as predictor variables. Indicator (binary) variables (value 0 or 1 ) for each element/module of the programme indicated the participation or not in the specific module and these were managing anxiety, managing depression, stress management, education, pain management, lifestyle management, assertiveness, confidence building, and managing fatigue. Elements poorly attended, were omitted from the analysis. The outcome variable was completion (dichotomous: completed/discharged); and for those who completed the difference in pre- and post-programme HADS scores.

Univariate analysis on the performance measures was completed to investigate differences in pre- and post-programme HADS values. A general linear model examined which demographic variables were significant predictors of the degree of change in the performance measure, with a backward stepwise method used to determine the best model.

## Results

The first client was referred to the programme in July 2006 and on average there were 112 referrals per month, totalling 2016 referrals during the study’s evaluation period. The gender distribution of the client group was evenly split, with 1014 (50.3%) females and 1002 (49.7%) males. Client age ranged from 18.1 to 65.8 years-old. Mean age at referral was 40.5±11.3 years, with no statistically significant difference between the mean female (40.9±11.3) and male ages (40.1±11.3) (t=1.6, df=1877, ns).

The results demonstrated that 98% of clients were allocated to practitioners within 5 working days of referral and almost 99% of clients were allocated within 7 working days. Of the 1,672 referrals for which a first appointment was arranged, 678 (40.6%) first appointments exceeded the 10 working-day limit. The main stages of withdrawal from the programme were clients not attending the first agreed upon appointment (21.4%), clients not proceeding with the CMP after their initial assessment (15.3%), and clients discharged having completed only some of the planned activity (15.6%).

The majority (62.8%) of clients had a mental-health condition (Table 1) as a primary health condition. A higher proportion of females (66.1%) presented mental-health problems compared to males (59.4%), while the opposite was observed for musculoskeletal problems (22.6% versus 30.2%, respectively (χ^2^=22.7, df=3, *p*<001).

**Table 1:** **Primary health condition by gender** (**N**= **1**,**906**)

**Health condition**	**Gender**		**Total**
	**Male**	**Female**	
Mental Health	562 (59.4%)	635 (66.1%)	1197 (62.8%)
Cardiorespiratory	38 (4.0%)	22 (2.3%)	60 (3.1%)
Musculoskeletal	286 (30.2%)	217 (22.6%)	503 (26.4%)
Other	60 (6.3%)	60 (6.3%)	146 (7.7%)
**Total**	**946** (**100**%)	**960** (**100**%)	**1906** (**100**%)

The mean number of sessions clients attended with a Condition Manager was 6.5±2.6 (min=1, max=17), while the mean number of weeks in the programme was 11.6±4.3 (min=1, max=25). Clients participated in 3.3±1.4 elements (min=1, max=9). The majority of clients participated in the “managing anxiety” element (62.8%), and just over half participated in the “managing depression” element (50.8%). Confidence building (40.4%), stress management (33.2%), lifestyle management/health promotion (31.4%), pain management (30.2%) and education on health condition (27.9%) were other elements well attended.

Successful completion was 28.5% (575 “completers”; 144 “discharges”). Amongst completers, 6 clients completed following their initial assessment appointment, whilst 569 completed after having more than one appointment with a Condition Manager. A statistically significantly higher proportion of clients within South Lanarkshire (32.2%) were classified as “completers” compared to North Lanarkshire (25.5%) (χ^2^=1.1, df=1, *p*<0.001). Completion of the programme by males (27.4%) and females (29.6%) was not significantly different. Clients over 40-years had significantly higher completion percentages (35.4%) compared with clients under 40-years (20.9%) (χ^2^=51.0, *p*<0.001). There was a statistically significantly (*p*<0.001) lower completion amongst clients presenting with mental health issues (25.5%) compared to cardiorespiratory (33.9%), musculoskeletal (33.2%) or other issues (35.5%).

Just under two thirds (65.5%) of the client phone numbers supplied were landlines and 34.5% were mobile numbers. Completion percentages of mobile-phone users (18.3%) and landline (33.5%) users were statistically different. Amongst clients under 40-years, completion for mobile users was significantly lower (13.9%) than for landline users (25.4%) (χ^2^=15.9, df=1, *p*<0.001). Similar results were observed for clients over 40-years, mobile users showed a statistically significant lower completion (23.9%) compared with landline (39.1%) users (χ^2^=18.3, df=1, *p*<0.001).

Whether clients completed, refused to commence or “dropped-out” of the programme, they could still be re-referred up to a maximum of three times. Therefore, the database contains 2016 referrals, but only 1906 clients (98 clients were referred twice, 6 clients were referred three times). Of the 98 clients referred twice, 69.4% failed to complete the programme on either occasion, 21.4% completed following their second referral after being discharged on the first, 5.1% completed on both occasions, and 4.1% were discharged on the second occasion, following successful completion on the first. All 6 clients who were referred to the project 3-times failed to complete the programme on every occasion.

Of the 569 clients who successfully completed the CMP programme attending more than one appointment, 267 (47%) completed at least part of the Client’s Feedback Form. Overall, clients had a positive view of the programme, with both the programme (98%) and practitioners (99.6%) receiving “excellent” scores by 98% and 99.6% of the clients, respectively. The overwhelming response was that the clients felt that taking part in the programme was worthwhile (95%), it made them feel more confident (91%) and would recommend it to others (99%).

Collectively, 25 CMP practitioners were employed in North and South Lanarkshire during the study period. Completion by practitioners varied between 0–44.6%. The lowest number of clients allocated to a practitioner was 4, and the highest 162. No evidence of an association was observed between practitioner experience, defined as number of clients seen, and client completion percentages.

Practitioners and clients were categorised into the same four age and gender groups (cut-off of 40-years of age) and, logistic regression was applied. The number of referrals and associated completion percentages between the four client/practitioner categories demonstrated that amongst younger-males completion is higher, if the practitioner is a young-male or young-female (Table 2). In these permutations, the respective completion percentages (28.0% and 27.9%) approximate the overall completion percentage of 28.5%. For older-males, completion is highest if they interact with older-male practitioners. However, there is no statistical significance in this group. Young-females manifest statistically significant higher-completion percentages if seen by a female practitioner, while older-females show low completion if seen by young-female practitioners.

**Table 2:** **Number** (**N**) **of client**/**practitioner permutations and associated percentages of completion** (**C**) **by age**/**gender and occupational background of practitioner and primary presenting issue** (**N**_**practiioners**_= **25**; **N**_**clients**_= **1**,**989**)

		**Clients**									
		**Younger males**		**Older males**		**Younger females**		**Older females**		**Total**	
**Practitioners (**^**a**^**)**		N (−)	C (%)	N (−)	C (%)	N (−)	C (%)	N (−)	C (%)	N (−)	C (%)
	younger males	50	28.0	64	32.8	32	12.5	57	38.6	203	30.0
	older males	75	18.7	60	40.0	32	15.6	46	43.5	213	29.6
	younger females	147	27.9	153	34.6	151	22.5	159	23.9	610	27.2
	older females	227	13.2	211	35.5	229	24.0	293	39.9	960	28.9
	Total	499	19.8	488	35.5	444	22.1	555	35.5	1986	28.5
	Significance^**1**^		**		**ns**		**ns**		**		*
		**Primary Condition**									
		**Cardio**- **respiratory**		**Mental Health**		**Musculo**- **skeletal**		**Other**		**Total**	
**Background (**^**b**^**)**		**N (−)**	**C (%)**	**N (−)**	**C (%)**	**N (−)**	**C (%)**	**N (−)**	**C (%)**	**N (−)**	**C (%)**
	Mental Health	25	52.0	621	28.2	171	35.1	62	43.5	879	
	Physiotherapy	7	28.6	77	15.6	144	30.6	20	35.0	248	
	General	16	18.8	306	27.1	132	37.9	42	33.3	496	
	Learning Disability	13	23.1	278	20.9	71	25.4	28	21.4	390	
	Total	61	34.4	1282	25.6	518	33.2	152	35.5	2013	
	Significance^**1**^		-		*		**ns**		**ns**		

**(**^**a**^**)Note**: 30 client/practitioner permutations are missing. ^**1**^: *=p<0.05, **=p<0.01, ***=p< 0.001. ns=not significant.

**(**^**b**^**)Note**: 3 permutations are missing; ^**1**^ *=p<0.05, **=p<0.01, ***=p<0.001; ns = not significant.

The 25 practitioners were assigned to one of four occupational backgrounds, i.e. mental health (32%), learning disabilities (28%), physiotherapy (16%), and general background (24%). Clients could have multiple health conditions; however, one is designated as their primary condition (Table 1). For the primary condition of mental-health (Table 2), logistic regression demonstrated that higher-completion was observed when practitioners had either a mental-health or general background (χ^2^=9.9, df=3, p<0.05). For musculoskeletal and other issues, there was no evidence of an association with practitioner’s background. Due to the small number of clients presenting with cardiorespiratory issues, a formal χ^2^ test was invalid.

Forward stepwise logistic regression was applied with discharge as the dependent variable and the following independent variables: age, gender, depcat, condition, phone type, area, Jobcentre Plus, and practitioner. The number of clients covered by the analysis was *n* = 1,876 (missing data for n=30). The variables age, phone type, practitioner (all at *p*<0.001) and condition (*p*=0.028) were found to be significant predictors. The Hosmer-Lemeshow goodness-of-fit test suggested that the model was acceptable. The analysis (Table 3) demonstrated that a client is more likely to be discharged and not complete the CMP if he/she is younger, supplied a mobile telephone number, or had a mental health condition.

**Table 3:** **Odds ratio of being discharged from the CMP** (**N**=**1**,**876**)

**Variable**	**Odds ratio**	**95%CI lower bound**	**95%CI upper bound**	***p***-**value**
Age (one year increase)	0.970	0.960	0.979	<0.001
Condition (mental health vs. physical health)	1.34	1.04	1.71	0.028
Phone type (mobile vs. landline)	2.02	1.55	2.62	<0.001

**Figure 1:**
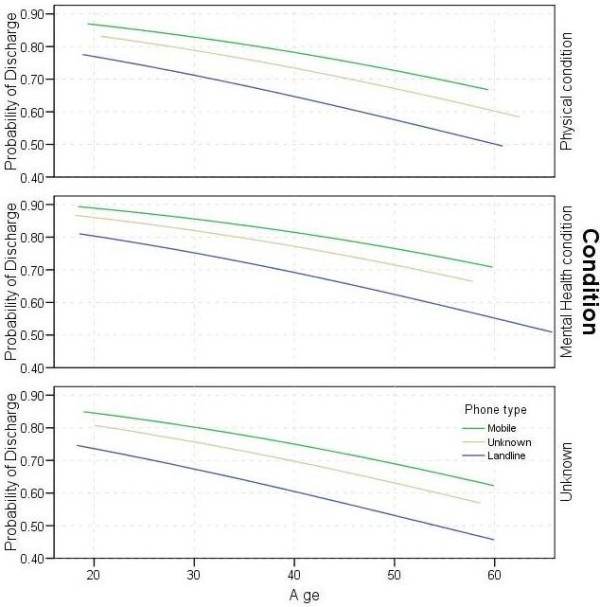
**Predicted probabilities of client being discharged with a given age**, **condition and phone type.**

Running the analysis including HADS Anxiety and Depression pre-intervention scores as independent variables decreased the number of included clients from 1,876 to 827. The HADS scores were not statistically significant, but age, phone type, condition and practitioner were still significant. The logistic regression was performed again, using age, condition and phone type as predictor variables. For simplicity the model assumed an “average” practitioner. Estimates varied from under 50% for a 60-year old client with a physical-health condition and landline telephone, to nearly 90% for a 20-year old client with a mental-health condition and mobile telephone (Figure 1).

### Performance measures

Overall, 838 measures of anxiety and 839 measures of depression were recorded at the pre- assessment stage (Table 4), with a mean anxiety score of 12.49±4.53 (min=0, max=21) and a mean depression score of 9.84±4.23 (min=0, max=21). This includes measures of both clients who completed and were discharged from the programme.

**Table 4:** **Categories of pre**- **and post assessment anxiety and depression** (**N**_**HADSanxiety**_=**838**; **N**_**HADSdepression**_=**839**)

**Pre**-**assessment**				
	**Normal**	**Mild**	**Moderate**	**Severe**
Anxiety	136 (16.2%)	120 (14.3%)	346 (41.3%)	236 (28.2%)
Depression	266 (31.7%)	198 (23.6%)	288 (34.3%)	87 (10.4%)
**Post**-**assessment**				
	**Normal**	**Mild**	**Moderate**	**Severe**
Anxiety	203 (43.9%)	117 (25.3%)	110 (23.8%)	32 (6.9%)
Depression	322 (69.7%)	73 (15.8%)	59 (12.8%)	8 (1.7%)

Table 4 shows that 69.5% of the clients would be considered anxious and 44.7% of clients depressed pre-assessment. Furthermore, 73.6% of clients were either anxious, depressed or both pre-assessment. A significantly higher proportion of clients presenting with mental-health issues were considered anxious (80.5%) (χ^2^=74.8, df=3, *p*<0.001) and depressed (52.4%) (χ^2^=32.4, df=3, *p*<0.001), than all other health conditions. Post-assessment, 462 measures of anxiety and depression were recorded (Table 4), with a mean anxiety score of 8.69±4.12 (min=0, max=21) and 5.77±4.16 (min=0, max=18) for mean depression. Overall, 30.7% of clients would be considered anxious and 14.5% depressed post-assessment. Furthermore, 32.5% of clients were either anxious, depressed or both post-assessment. If one considers the clients with a physical-health condition only, the reductions in both anxiety and depression are still statistically significant in both cases (*p*<0.001). The mean reductions are 2.25 and 2.89, respectively. This compares to mean reductions of 4.74 (anxiety) and 4.72 (depression) for clients with a mental-health condition.

In total, 457 clients provided anxiety scores and 458 clients provided depression scores at both pre- and post-assessment. There was a statistically significant decrease in the mean scores of both anxiety (*t*=21.7, df=456, *p*<0.001) and depression (*t*=21.0, df=457, *p*<0.001). Of the 328 clients who were anxious pre-assessment, 197 (60.1%) were not considered anxious post-assessment (Table 5). A statistically significant change towards being more anxious was recorded for 8 clients (*p*<0.001). Of the 202 clients who were depressed pre-assessment, 146 (72.3%) were not depressed post-assessment. A statistically significant change towards being more depressed was recorded for 10 clients (*p*<0.001).

**Table 5:** **Changes in the severity in anxiety and depression**: **pre**- **and post**- **assessment** (**N**_**HADSanxiety**_=**457**; **N**_**HADSdepression**_=**458**)

	**Post**-**Assessment**	
**Pre**-**Assessment**	**Not Anxious**	**Anxious**
Not Anxious	121 (93.8%)	8 (6.2%)
Anxious	197 (60.1%)	131 (39.9%)
	**Post**-**Assessment**	
**Pre**-**Assessment**	**Not Depressed**	**Depressed**
Not Depressed	246 (96.1%)	10 (3.9%)
Depressed	146 (72.3%)	56 (27.7%)

### General linear model

While 549 clients completed the programme on first referral, complete data for HADS Anxiety scores was for 448 clients, and 425 for HADS Depression scores. A general linear model was fitted with the difference in HADS Anxiety as the dependent variable; Age, Sessions, Weeks and Elements as covariates; and Gender, Phone type, Depcat, Condition, Area, and the indicator variables for each element of the programme, as fixed factors. The Practitioner and Jobcentre Plus variables, were left out to avoid small cell sizes. A backward stepwise method was used, and at each step, the least significant variable was eliminated, until all the remaining variables had significance of less than 0.05. In the final model, only the variables Condition and Managing Anxiety were retained (*p*=0.03 and *p*<0.001, respectively). The parameter estimates (Table 6) demonstrate that a mental-health condition is associated with an estimated reduction in HADS Anxiety of 1.11 in excess of that for a physical-health condition, and that attending the Managing Anxiety module is associated with an estimated reduction in HADS Anxiety of 1.57 greater than that achieved by not attending this module.

**Table 6:** **Parameter estimates**, **significance**, and confidence intervals for HADS anxiety* (**upper**; **N**=**448**) and depression** (**lower**; **N**=**425**)

**HADS Anxiety**	**Variable**	**Estimate**	**Significance** (***p***-**value**)	**95%CI lower bound**	**95%CI upper bound**
	Condition	−1.11	0.03	−2.10	−0.12
	Managing Anxiety	−1.57	0.001	−2.29	−0.75
**HADS Depression**	**Variable**	**Estimate**	**Significance** (***p***-**value**)	**95%CI lower bound**	**95%CI upper bound**
	Managing Depression	−1.28	0.002	−2.09	−0.47
	Managing Anxiety	−1.33	0.002	−2.18	−0.48
	Sessions	−0.226	0.011	−0.40	−0.05
	Weeks	+0.126	0.025	0.016	0.236

* Pre-assessment HADS anxiety: 12.49±4.53; Post-assessment HADS anxiety: 8.69±4.12.

** Pre-assessment HADS depression: 9.84±4.23; Post-assessment HADS depression: 5.77±4.16.

The general linear model analysis was repeated using the difference in HADS Depression as the dependent variable (Table 6). This demonstrated that attendance of the modules Managing Depression and Managing Anxiety were associated with a reduction in HADS-Depression score of 1.28 and 1.33 respectively, compared to non-attendance. The HADS-Depression score was reduced by 0.23 for every additional session attended, but it increased by 0.13 for every additional week spent on the programme.

## Discussion

The results demonstrated that during the study investigation period the majority of the clients presented with a mental-health condition. Overall, 28.5% of clients successfully completed the programme. Several factors, such as age, health condition, mode of contact and practitioner characteristics, were significant determinants of outcome. The age/gender synthesis of the client/practitioner pair was significantly important in most of the combinations, whereas a practitioner's background was only important in the case of mental health. The results showed that participation in the CMP was associated with a better health status, by reducing by more than 50% the clients that were either anxious, depressed or both before undertaking the programme, i.e. from 74% to 32.5%.

The key question that emerges is why is completion so low and discharge so high (72%)? The majority of the clients that completed that programme felt that taking part was worthwhile, made them feel more confident and would recommend it to others. This is in line with previous studies, which also reported general satisfaction from the patient perspectives of CMP programmes and that patients experienced improved health behaviours and psychosocial outcomes [27-31]. Previously, clients reported concerns over the duration and accessibility of the interventions, and recommended that the dynamics of the situation and the characteristics of the individuals should be accounted for when designing and offering the services [27]. One way the Lanarkshire CMP have attempted to achieve greater accessibility to the service is by conducting the majority of the intervention remotely by telephone. Telephone-based interventions are also being used successfully for other chronic disorders [32-34], and while personal contact with health professionals is still a preferred method, there is change towards communication via other routes, for example the internet [32,35], which further supports the tele-based implementation of intervention programmes. This could also provide the opportunity for other health professionals to exploit these means of contact and information provision when developing information and advisory services.

Our findings showed that an individual's characteristics greatly influence whether or not they will be successful or not in the programme. A "selection effect" and a "duration effect" have been reported to assess why some people remain on benefits for longer periods of time than others [36]. The former hypothesizes that some claimants have characteristics which enable them to leave benefits quickly and others have a permanently low chance of recovering their health and/or getting a job [36]. The latter is based on the fact that the longer one remains on benefit, the lower their chances of leaving. [36]. This effect was addressed in the CMP and that is why it tried to intervene at a very early stage of going on benefits. However, further research would need to assess the optimum time in the benefit-claiming cycle one must intervene to receive maximum output in terms of overall wellbeing and return-to-work, but generally it is believed that earlier interventions are beneficial [9].

Concerning the selection effect, our study demonstrated that an individual's characteristics, the implemented programme and the interaction and inter-relationships that evolve during this process can influence outcome and this is further supported in the literature [2,10,37,38].

The matching of the client and practitioner was seen as an important determinant. While, it was not the experience in terms of client number each practitioner had dealt with, it was the background of each practitioner and the way the client and practitioner matched in terms of age and sex that was important. This phenomenon of client-practitioner characteristics predicting intervention outcome is not limited to vocational rehabilitation, but has been observed in other interventions as well [39]. While in some cases the matching of the pair was counterproductive, this knowledge of an important association could be useful and beneficial in assigning clients to practitioners not only on a skill match basis but using the predictors of age and sex to maximise successful completion for clients.

Clients referred to the programme can present with multiple health conditions, one of which is designated as their primary condition. One might have expected that the programme would reduce the HADS scores of mental health clients, but make no difference to physical health clients. However, it is known that clients are often co-morbid, for example suffering both physical and mental health problems. Additionally, the results showed that the HADS-depression score was reduced for every additional session a client attended, however, increased for every additional week a client spent on the programme. This result could be a short-term ‘novelty effect’, and it may still be that longer attendance on the programme has better long-term outcomes, especially as patients have expressed concerns over the short duration of the CMP programme [27]. However, it should be noted that the estimated effect is small. A difference of ten weeks would only produce a difference in scores of around 1.3. Another possible cause of this counter-intuitive result may be the relatively high correlation between the variables sessions attended and weeks on the programme.

Public policy in the UK through Welfare to Work is increasingly using a variety of interventions similar to that in the *Condition Management Programme*[40]. One such example is the new *Work Programme*, which was introduced in April 2011 to replace the PtW scheme [14]. While this study focuses on a voluntary programme, which may have influenced participation and motivation, it provides some evidence that the systematic evaluation of these programmes could identify ways in which other similar interventions could be improved. No occupational outcomes were available in this analysis, because of the data disconnect between the CMP and other Government agencies. Vocational outcome data would have been very worthwhile in this study and proper record linkage is recommended for future programmes. To improve the efficiency of these programmes it is essential that lessons are learned and implemented [41,42]. Analyzing the effectiveness of the intervention programmes could also be of benefit to practitioners. It provides more detail on the factors that influence whether certain interventions are beneficial or not depending on personal characteristics. However, the availability of reliable and accessible longitudinal data is necessary in this field. In general, there is a dearth of data on the relative long-term effectiveness of different programmes and services, measured by tracking the employment and benefits status of claimants over time. In the US [43,44] some attempts have been made to look at the cost effectiveness of supported employment programmes, and similar work has been undertaken in the UK [45]. Some US work is attempting to develop longitudinal analysis [43], but further development of such methodologies would be valuable [5]. A consistent method of recording performance measures, data validation, and conditional formatting could be utilised in real settings to improve recording of the data that could provide the evidence-base for more tailored and appropriate services for a client group.

## Conclusion

Our study demonstrated an association of a telephone-based biopsychosocial intervention with improved health for those receiving Incapacity Benefit. Client and practitioner characteristics, such as age, gender, health condition, as well as mode of contact were significant determinants of outcome. The results showed that participation in the CMP was associated with a better health status, by reducing by more than 50% the clients that were either anxious, depressed or both before undertaking the programme. This study provides evidence that the systematic evaluation of such programmes and interventions could identify ways in which they could be improved.

## Abbreviations

IB: Incapacity benefit; PtW: Pathways to work; CMP: Condition management programme; NHS: National Health Service; CBT: Cognitive behavioural therapy; HADS: Hospital anxiety and depression scale; DepCat: Deprivation category.

## Competing interests

The authors declare they have no competing interests.

## Authors’ contributions

ED was the main author of the manuscript and substantially contributed to the interpretation of the data. IG carried out the statistical analysis and drafted sections of the manuscript. EBM conceived the study and participated in its coordination. All authors read and approved the final manuscript.

## Pre-publication history

The pre-publication history for this paper can be accessed here:

http://www.biomedcentral.com/1471-2458/12/927/prepub
